# Docetaxel Rechallenge vs Cabazitaxel in Patients With Metastatic Castration-Resistant Prostate Cancer

**DOI:** 10.1001/jamanetworkopen.2025.51231

**Published:** 2026-01-16

**Authors:** Pedro C. Barata, June K. Corrigan, Jennifer La, John M. Culnan, Elliot Akama-Garren, Karlynn N. Dulberger, Clark Dumontier, Jason Hansen, John R. Bihn, Rhonda L. Bitting, Mary T. Brophy, Heather H. Cheng, Matthew R. Cooperberg, Nhan V. Do, Tanya Dorff, Antonio Tito Fojo, J. Michael Gaziano, Sergey D. Goryachev, Susan Halabi, Richard L. Hauger, David M. Nanus, Timothy R. Rebbeck, Chong-Xian Pan, Martin W. Schoen, Kaitlin N. Swinnerton, Kenute Myrie, Rachel B. Ramoni, Nathanael R. Fillmore, Channing J. Paller, Matthew B. Rettig

**Affiliations:** 1 Division of Solid Tumor Oncology, Department of Medicine, University Hospitals Cleveland Medical Center, Case Western Reserve University School of Medicine, Cleveland, Ohio; 2 Massachusetts Veterans Epidemiology Research and Information Center, Department of Veterans Affairs Healthcare System, Boston; 3 Department of Medicine, Harvard Medical School, Boston, Massachusetts; 4 Section of Hematology and Medical Oncology, Boston University School of Medicine, Boston, Massachusetts; 5 Department of Medicine, Brigham and Women’s Hospital, Boston, Massachusetts; 6 College of Osteopathic Medicine, University of New England, Biddeford, Maine; 7 Durham Veterans Affairs (VA) Medical Center, Durham, North Carolina; 8 Department of Medicine, Duke University School of Medicine, Durham, North Carolina; 9 Division of Oncology, Department of Medicine, University of Washington, Seattle; 10 Clinical Research Division, Fred Hutchinson Cancer Research Center, Seattle, Washington; 11 Surgical Service, San Francisco VA Medical Center (VAMC), San Francisco, California; 12 Department of Medical Oncology and Therapeutics Research, City of Hope Comprehensive Cancer Center, Duarte, California; 13 Herbert Irving Comprehensive Cancer Center, Columbia University and James J. Peters VAMC, Bronx, New York; 14 Research Service, Department of Veterans Affairs San Diego Healthcare System, San Diego, California; 15 Center for Behavior Genetics of Aging, School of Medicine, University of California, San Diego, La Jolla; 16 Department of Medicine, Weill Cornell Medicine, New York, New York; 17 Harvard T. H. Chan School of Public Health, Cambridge, Massachusetts; 18 Dana-Farber Cancer Institute, Boston, Massachusetts; 19 John J. Cohran Veterans Hospital, VA St Louis Healthcare System, St Louis, Missouri; 20 Department of Medicine, St Louis University School of Medicine, St Louis, Missouri; 21 Office of Research and Development, Veterans Health Administration, US Department of Veterans Affairs, Washington, DC; 22 Department of Oncology, Johns Hopkins University School of Medicine, Baltimore, Maryland; 23 Division of Hematology-Oncology, Greater Los Angeles Veterans Affairs Medical Center, Los Angeles, California; 24 Prostate Cancer Program of the Institute of Urologic Oncology, University of California, Los Angeles, Los Angeles

## Abstract

**Importance:**

Docetaxel has been a standard treatment for metastatic castration-resistant prostate cancer (mCRPC) since 2004. Cabazitaxel, a related taxane, was approved in 2010 for patients with mCRPC who had been previously treated with docetaxel. The comparative effectiveness of docetaxel rechallenge vs switching to cabazitaxel after prior docetaxel for mCRPC remains unclear.

**Objective:**

To compare the clinical outcomes associated with docetaxel rechallenge vs cabazitaxel in patients with mCRPC who did not experience disease progression during prior administration of docetaxel in the mCRPC setting.

**Design, Setting, and Participants:**

This retrospective cohort study was conducted in the nationwide Veterans Affairs health care system, using inverse probability of treatment weighting to control for potential confounders. Patients who were diagnosed with chemonaive mCRPC between January 1, 2010, and December 31, 2023, received initial docetaxel treatment, and did not experience disease progression were eligible to participate.

**Exposures:**

Treatment with docetaxel rechallenge or cabazitaxel.

**Main Outcomes and Measures:**

The primary outcome was overall survival (OS) from the initiation of the second course of taxane, which was compared in patients treated with docetaxel rechallenge vs cabazitaxel using weighted Kaplan-Meier analysis and Cox proportional hazards regression models. Secondary outcomes included prostate-specific antigen response, time to next systemic treatment or death, and subsequent treatments received.

**Results:**

A total of 669 patients (407 receiving cabazitaxel and 262 receiving docetaxel rechallenge) with a median age of 72 (IQR, 67-77) years were included. Patients treated with docetaxel rechallenge had a significantly longer OS (median, 12.3 [IQR, 10.5-13.8] months) compared with those treated with cabazitaxel (median, 9.6 [IQR, 8.6-11.1] months), with a hazard ratio of 0.81 (95% CI, 0.55-0.99; *P* = .04). Descriptive analysis of secondary outcomes was consistent with this finding, including prostate-specific antigen response (weighted 9.8% achieving reduction of 90% or more in the docetaxel rechallenge group vs 3.0% in the cabazitaxel group) and time to next treatment or death (median, 10.7 [IQR, 7.8-12.7] months in the docetaxel rechallenge group vs 8.9 [IQR, 7.1-10.5 months] in the cabazitaxel group). Use of platinum, immunotherapy, or poly (ADP-ribose) polymerase inhibitors was similar between patients treated with cabazitaxel and docetaxel rechallenge.

**Conclusions and Relevance:**

In this cohort study of patients with mCRPC, docetaxel rechallenge was associated with improved OS compared with cabazitaxel among patients who did not experience disease progression during prior docetaxel for mCRPC. These findings support docetaxel rechallenge as a treatment option for patients in this scenario.

## Key Points

**Question:**

Are there different outcomes associated with docetaxel rechallenge and cabazitaxel in patients who received prior docetaxel for metastatic castration-resistant prostate cancer (mCRPC) and did not experience disease progression?

**Findings:**

In this cohort study of 669 patients in the nationwide Veterans Affairs health care system, docetaxel rechallenge was associated with longer overall survival compared with cabazitaxel.

**Meaning:**

Given that randomized clinical trials are unlikely to be conducted for this scenario, these findings support docetaxel rechallenge for patients with mCRPC who did not experience disease progression during prior docetaxel treatment.

## Teaser

This cohort study compares overall survival in patients with metastatic castration-resistant prostate cancer (mCRPC) receiving cabazitaxel or docetaxel rechallenge after initial treatment with docetaxel.

## Introduction

Prostate cancer is the second leading cause of cancer-related mortality in the US. Metastatic castration-resistant prostate cancer (mCRPC) accounts for the overwhelming majority of deaths attributable to prostate cancer.^1^ During the last 20 years, numerous therapies, including cytotoxic chemotherapy, androgen receptor pathway inhibitors (ARPI), radioligands, and others, have received regulatory approval for treatment of mCRPC.^2^ However, the clinical use of these therapies is heterogenous, and the optimal sequencing of these agents is not well defined.^3^

In 2004, docetaxel, a semisynthetic taxane, was the first drug to receive regulatory approval for mCRPC based on improved overall survival (OS).^4^ More recently and relative to the impact of docetaxel used in the mCRPC setting, docetaxel has been demonstrated to achieve more marked improvement in OS when added to the backbone of androgen deprivation therapy in the setting of metastatic hormone-sensitive prostate cancer (mHSPC).^5^ In principle, patients who have received docetaxel for mHSPC can be rechallenged with docetaxel when they transition to mCRPC. In this context, docetaxel rechallenge has been shown to be well tolerated and effective.^6,7^

Cabazitaxel was approved in 2010 for patients with mCRPC who had previously received docetaxel based on its improved OS compared with mitoxantrone.^8^ Cabazitaxel is a demethylated derivative of docetaxel with higher cell penetration due to 2 methoxy groups on the taxane ring that increase its lipophilicity. Among patients who have received docetaxel and 1 ARPI, cabazitaxel improved OS compared with a second ARPI.^9^ Cabazitaxel is associated with more neutropenia and less neuropathy compared with docetaxel, while there was no difference in median OS when used as the first-line treatment for mCRPC in the FIRSTANA study.^10^

In clinical practice, it is common for patients with mCRPC to discontinue docetaxel treatment for reasons other than disease progression, such as to manage cumulative toxic effects, complete a planned course of therapy, or take a planned break in treatment after a durable response. For these patients, the optimal subsequent treatment is unclear, with options including both docetaxel rechallenge and cabazitaxel.

The importance of elucidating the clinical impact of the choice of the second taxane after initial docetaxel treatment for mCRPC is highlighted by the increased use of granulocyte colony-stimulating factor (G-CSF) with cabazitaxel and the markedly greater cost of cabazitaxel, which remains under patent protection, compared with docetaxel, which has been available as a generic drug for many years. According to pharmaceutical pricing data from the US Department of Veterans Affairs (VA) Office of Procurement, Acquisition and Logistics, the drug costs of a 6-cycle course of cabazitaxel (20 mg/m^2^) and docetaxel (75 mg/m^2^) are approximately $53 990 and $85, respectively.^11^

We leveraged our large clinical dataset in the VA, the largest integrated health care system in the US, to evaluate the relative impact of docetaxel rechallenge vs cabazitaxel for patients who had received docetaxel as their first instance of a taxane for mCRPC and discontinued docetaxel treatment without evidence of progression. Our primary objective was to compare OS in patients with mCRPC treated with cabazitaxel or docetaxel rechallenge after previous treatment with docetaxel in the mCRPC setting (as opposed to mHSPC). We hypothesized that cabazitaxel would be more effective than docetaxel rechallenge in patients with mCRPC who had previously been treated with docetaxel, as measured by OS.

## Methods

### Study Design, Data Sources, and Participants

This retrospective cohort study compared outcomes of patients in the nationwide VA health care system who received initial docetaxel treatment for mCRPC, and later, among those without disease progression, docetaxel rechallenge or cabazitaxel. Data were obtained from the VA Corporate Data Warehouse (CDW), which collates administrative and electronic health record data from VA facilities throughout the US. This study was approved by the VA Boston Healthcare System Research and Development Committee as an exempt study prior to data collection and analysis, with a waiver of informed consent per the Common Rule due to use of existing data. The study followed the Strengthening the Reporting of Observational Studies in Epidemiology (STROBE) reporting guideline.

Patients were diagnosed with chemonaive mCRPC between January 1, 2010, and December 31, 2023, then received a course of treatment with docetaxel of at least 3 cycles and later received a second course of treatment (docetaxel rechallenge or cabazitaxel) at least 3 months after the last dose of the first course of docetaxel. Patients did not receive chemotherapy between their initial course of docetaxel and docetaxel rechallenge or cabazitaxel. Combination regimens including other agents such as carboplatin were allowed. Patients who received etoposide—the use of which is associated with neuroendocrine or aggressive phenotype prostate cancer—after mCRPC diagnosis were excluded, as were patients with disease progression during initial docetaxel treatment, which was defined using prostate-specific antigen (PSA) criteria only as an increase in PSA level of 25% or more. Patients were also excluded if they lacked a baseline PSA value within 1 year before index date.

Treatments given in inpatient and outpatient settings at the VA, as well as claims for outside care paid for by the VA, were captured. Date of mCRPC diagnosis was defined as the later of the dates associated with CRPC or metastatic prostate cancer diagnoses, as captured in the VA Informatics and Computing Infrastructure Prostate Data Core. Date of metastatic prostate cancer diagnosis was identified using a natural language processing algorithm on radiology, urology, and oncology notes, with specificity of 97.9% and sensitivity of 91.9%.^12^ CRPC diagnosis date was identified using natural language processing on clinical notes in combination with structured laboratory and medication data, with sensitivity of 97.9% and specificity of 99.2%.^13^

### Outcomes and Covariates

The primary outcome was OS, defined as the time from the index date to death due to any cause. The index date was the date of the start of the second course of taxane treatment. Patients were censored at the end of the study period, December 31, 2023, if they were still alive. Death date was obtained from vital status information in the VA CDW (which provides 98.3% sensitivity and 97.6% exact agreement against the US National Death Index); therefore, it was unnecessary to censor for loss to follow-up.^14^

Secondary outcomes included PSA response, time to next systemic treatment or death, and subsequent treatments received. PSA response was defined as the proportion of patients achieving a reduction of a certain percentage from the PSA level measured at baseline. Time to next systemic treatment or death was defined as time from the index date to a new systemic prostate cancer treatment, not including castration or death. Patients were censored at the end of the study period or at the first break in continuous follow-up after the index date, defined as the end of a 90-day gap without any clinical encounter at the VA or the last PSA laboratory test result. Subsequent treatments received were tabulated.

Baseline covariates were defined based on data recorded prior to or on the index date. Age and self-reported race and ethnicity were obtained using structured data in the VA CDW. Race and ethnicity were included because they were potential confounders, and the following categories were used: Black, Hispanic, non-Hispanic White, and other or unknown race and ethnicity (including American Indian or Alaska Native, Asian, Native Hawaiian or Other Pacific Islander, declined to answer, and unknown). Frailty was measured using the VA Frailty Index,^10^ and individual comorbidities were measured using definitions from the Centers for Medicare & Medicaid Services Chronic Conditions Warehouse, based on both diagnosis and procedure codes recorded in the 3 years prior to the index date. Prostate treatment information and PSA results were obtained from structured drug and laboratory data. Metastatic status and Gleason score at initial diagnosis, as well as prostatectomy or radiation therapy prior to index date, were obtained from the Prostate Data Core. Metastatic status was considered synchronous if patients had stage IV cancer at time of initial diagnosis, and metachronous otherwise. Cancer stage (N1, M1a, M1b, and M1c) at both the date of the first taxane treatment and the index date (date of the second taxane treatment) was obtained through manual review of text pertaining to metastatic status in clinical notes and radiology reports.

### Statistical Analysis

Inverse probability of treatment weighting (IPTW) was used to balance potential confounders in patients treated with docetaxel rechallenge and cabazitaxel. This IPTW approach was designed to emulate a randomized clinical trial, balancing patient characteristics to isolate the outcomes associated with the treatment choice itself. Potential confounders included age, race and ethnicity, comorbidities (Alzheimer disease, anemia, chronic kidney disease, cardiovascular disease, diabetes, and liver disease or viral hepatitis), frailty, PSA level at index date, PSA level doubling time at index date, PSA response to initial docetaxel treatment, Gleason score at initial prostate cancer diagnosis (dichotomized as above or below median score among the cohort), laboratory test results at index date (lactate dehydrogenase, albumin, hemoglobin, and alkaline phosphatase levels), synchronous vs metachronous metastatic disease, stage (N1, M1a, M1b, or M1c) at initial docetaxel treatment and at index date, cycles of initial docetaxel treatment, time from metastatic diagnosis to the index date, time from docetaxel treatment to the index date, and number of systemic prostate cancer drugs given before, between, and after the 2 taxane therapies. Continuous variables such as PSA level doubling time were dichotomized to simplify implementation of IPTW, allowing estimation of propensity scores using a more parsimonious model; cut points were chosen based on clinical relevance and prior literature.^15^ The estimated probability (ie, propensity score) of a patient receiving docetaxel rechallenge vs cabazitaxel, given the patient’s characteristics at baseline, used multivariable logistic regression that included these potential confounders as covariates. The distribution of propensity scores was examined for overlap between the 2 treatment groups. The propensity scores were used to calculate stabilized weights for IPTW. After weighting, balance was inspected by tabulating weighted patient characteristics within the treatment groups and calculating the standardized mean difference across groups.

After IPTW, Kaplan-Meier analysis was used to estimate the survival function for each outcome, stratified by treatment group, and a Cox proportional hazards regression model was fit to estimate the hazard ratio for docetaxel rechallenge vs cabazitaxel. Median follow-up was determined using the reverse Kaplan-Meier approach. Two-sided *P* < .05 indicated statistical significance. All analyses were conducted using R, version 4.4.1 (R Project for Statistical Computing).

## Results

**Figure 1.**
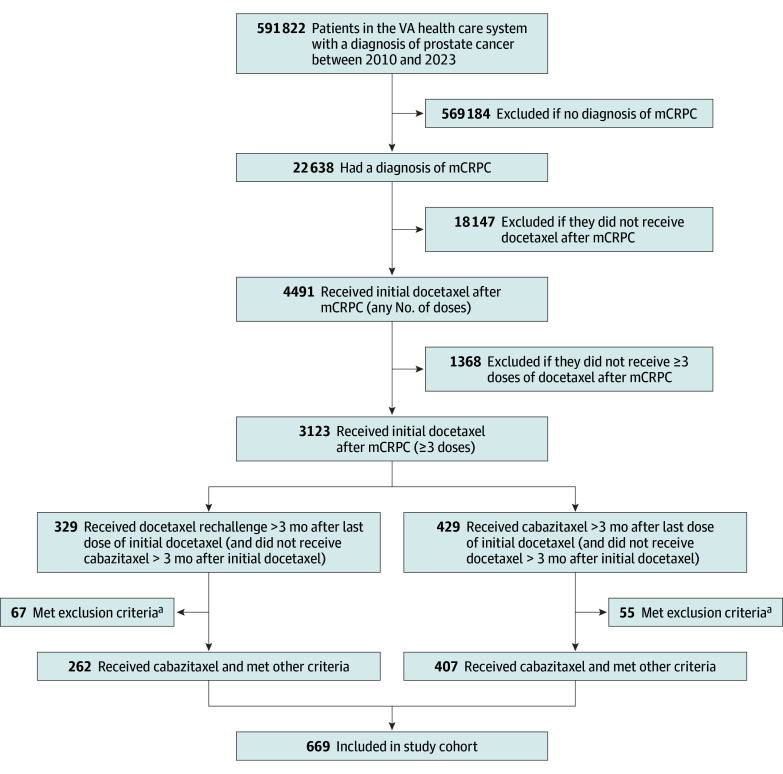
Patient Flow Diagram mCRPC indicates metastatic castration-resistant prostate cancer; VA, US Department of Veterans Affairs. ^a^Exclusion criteria consisted of receiving etoposide after mCRPC diagnosis, having no prostate-specific antigen (PSA) laboratory measures within 1 year before index date, or having evidence of rising PSA levels during initial docetaxel treatment (PSA level increase ≥25%).

We identified 669 patients meeting inclusion criteria (407 receiving cabazitaxel and 262 receiving docetaxel rechallenge) (Figure 1) with a median age of 72 (IQR, 67-77) years. In terms of race and ethnicity, 196 patients (29.3%) were Black, 29 (4.3%) were Hispanic, 407 (60.8%) were non-Hispanic White, and 37 (5.5%) were of other or unknown race or ethnicity. Baseline patient characteristics before and after IPTW are summarized in eTable 1 in Supplement 1 and the Table, respectively. Before weighting, patients treated with docetaxel rechallenge were older and had greater frailty. They also had lower median PSA levels at baseline, lower median Gleason scores, lower percentages of stage M1c cancer at the time of initial docetaxel treatment and at the index date, fewer median cycles of initial docetaxel treatment, and fewer systemic therapies between mCRPC diagnosis and index date and between initial docetaxel rechallenge and index date. After weighting, patient characteristics were well balanced (eFigure 1 in Supplement 1). The distribution of scores did not show evidence of nonpositivity or misspecification of the propensity score model (eFigure 2 in Supplement 1). The mean (SD) stabilized weight was 1.0 (0.7) (eFigure 3 in Supplement 1). The median follow-up time for overall survival was 13.7 (IQR, 12.3-15.4) months for group. Patients received a median of 3 (IQR, 1-3) prior systemic therapies. A weighted 125.9% (19.0%) of patients had a PSA response to initial treatment.

**Table.** Patient Characteristics in the Weighted Cohort^a^

Characteristic	Patients, weighted No. (%)			SMD
	Overall	Docetaxel rechallenge	Cabazitaxel	
Weighted No. of patients	662.4	265.2	397.2	NA
Age, median (IQR), y	72.21 (67.48-76.72)	72.29 (66.84-77.34)	72.16 (67.72-76.47)	0.013
Race and ethnicity				
Black	193.6 (29.2)	75.5 (28.5)	118.1 (29.7)	0.114
Hispanic	35.3 (5.3)	18.0 (6.8)	17.3 (4.4)	
Non-Hispanic White	399.6 (60.3)	156.8 (59.1)	242.7 (61.1)	
Other or unknown^b^	33.9 (5.1)	14.8 (5.6)	19.1 (4.8)	
Time of metastatic disease diagnosis				
Synchronous	220.0 (33.2)	84.8 (32.0)	135.2 (34.0)	0.045
Metachronous	257.9 (38.9)	105.8 (39.9)	152.1 (38.3)	
Unknown	184.5 (27.9)	74.6 (28.1)	109.9 (27.7)	
Stage at initial docetaxel treatment				
Pelvic lymph nodes only (N1)	25.1 (3.8)	10.4 (3.9)	14.7 (3.7)	0.075
Lymph nodes outside pelvis (M1a)	52.9 (8.0)	21.5 (8.1)	31.4 (7.9)	
Bone with or without lymph node (M1b)	509.4 (76.9)	205.7 (77.5)	303.8 (76.5)	
Visceral (liver, lung, or brain) with or without bone or lymph node (M1c)	62.8 (9.5)	22.1 (8.3)	40.8 (10.3)	
Unknown	12.1 (1.8)	5.6 (2.1)	6.5 (1.6)	
Stage at index date				
Pelvic lymph nodes only (N1)	14.4 (2.2)	6.5 (2.5)	7.8 (2.0)	0.087
Lymph nodes outside pelvis (M1a)	31.9 (4.8)	12.6 (4.8)	19.3 (4.8)	
Bone with or without lymph node (M1b)	504.9 (76.2)	204.0 (76.9)	300.9 (75.8)	
Visceral (liver, lung, or brain) with or without bone or lymph node (M1c)	107.5 (16.2)	39.8 (15.0)	67.7 (17.0)	
Unknown	3.7 (0.6)	2.3 (0.8)	1.5 (0.4)	
PSA level doubling time at index date				
≥3 mo	178.9 (27.0)	75.9 (28.6)	103.1 (25.9)	0.070
<3 mo	460.5 (69.5)	181.4 (68.4)	279.1 (70.3)	
Unknown	23.0 (3.5)	7.9 (3.0)	15.0 (3.8)	
PSA level at index date, median (IQR), ng/mL	81.35 (22.47-267.13)	94.49 (21.24-220.06)	79.77 (24.51-281.12)	0.054
Gleason score of median or above	599.2 (90.5)	238.6 (90.0)	360.6 (90.8)	0.029
No. of cycles of initial docetaxel treatment, median (IQR)	6.00 (4.00-10.00)	6.00 (4.00-9.00)	6.00 (4.00-10.00)	0.050
PSA response to initial docetaxel treatment				
≥50% Decline	125.9 (19.0)	48.9 (18.4)	77.0 (19.4)	0.051
≥70% Decline	66.6 (10.1)	27.8 (10.5)	38.8 (9.8)	
≥90% Decline	15.9 (2.4)	7.4 (2.8)	8.5 (2.2)	
Stable PSA level	454.0 (68.5)	181.1 (68.3)	272.9 (68.7)	
No. of systemic therapies before initial docetaxel treatment, median (IQR)	1.00 (0-2.00)	1.00 (0-2.00)	1.00 (0-2.00)	0.020
No. of systemic therapies between mCRPC diagnosis and index date, median (IQR)	2.00 (1.00-2.00)	2.00 (1.00-2.00)	2.00 (1.00-2.00)	0.040
No. of systemic therapies between taxane treatments, median (IQR)	1.00 (0-1.00)	1.00 (0-2.00)	1.00 (0-1.00)	0.100
Time from PC diagnosis to index date, median (IQR), mo	92.84 (50.51-151.02)	99.55 (50.79-150.74)	86.41 (50.37-150.27)	0.030
Time from initial docetaxel treatment to index date, median (IQR), mo	11.58 (8.17-18.00)	11.97 (8.26-18.70)	11.34 (8.15-17.36)	0.051
Comorbidities				
Alzheimer disease	1.2 (0.2)	0.0 (0.0)	1.2 (0.3)	0.078
Anemia	348.6 (52.6)	136.1 (51.3)	212.5 (53.5)	0.044
Chronic kidney disease	265.7 (40.1)	103.3 (39.0)	162.4 (40.9)	0.039
Cardiovascular disease	171.4 (25.9)	62.4 (23.5)	109.1 (27.5)	0.091
Diabetes	242.8 (36.7)	99.3 (37.4)	143.6 (36.2)	0.026
Liver disease or viral hepatitis	110.8 (16.7)	48.7 (18.4)	62.0 (15.6)	0.073
VA Frailty Index, median (IQR)^c^	0.23 (0.16-0.32)	0.23 (0.16-0.32)	0.26 (0.16-0.32)	0.098
Albumin level				
≥3.5 g/dL	415.8 (62.8)	162.6 (61.3)	253.2 (63.8)	0.108
<3.5 g/dL	197.9 (29.9)	78.6 (29.7)	119.3 (30.0)	
Unknown	48.7 (7.3)	24.0 (9.1)	24.7 (6.2)	
Hemoglobin level				
≥10 g/dL	494.0 (74.6)	199.9 (75.4)	294.2 (74.1)	0.033
<10 g/dL	151.5 (22.9)	58.5 (22.0)	93.0 (23.4)	
Unknown	16.8 (2.5)	6.9 (2.6)	10.0 (2.5)	
LDH level				
High	130.7 (19.7)	54.5 (20.6)	76.1 (19.2)	0.054
Not high	57.9 (8.7)	24.7 (9.3)	33.1 (8.3)	
Unknown	473.9 (71.5)	185.9 (70.1)	287.9 (72.5)	
Alkaline phosphatase level				
≥130 U/L	250.5 (37.8)	101.3 (38.2)	149.2 (37.6)	0.017
<130 U/L	392.9 (59.3)	156.1 (58.8)	236.8 (59.6)	
Unknown	19.0 (2.9)	7.8 (2.9)	11.2 (2.8)	

Abbreviations: LDH, lactate dehydrogenase; mCRPC, metastatic castration-resistant prostate cancer; NA, not applicable; PC, prostate cancer; PSA, prostate-specific antigen; SMD, standardized mean difference; VA, US Department of Veterans Affairs.

SI conversion factor: To convert albumin to g/L, multiply by 10; alkaline phosphatase to µkat/L, multiply by 0.0167; hemoglobin to g/L, multiply by 10; PSA to µg/L, multiply by 1.

^a^ Indicates after inverse probability of treatment weighting.

^b^ Includes American Indian or Alaska Native, Asian, Native Hawaiian or Other Pacific Islander, declined to answer, and unknown.

^c^ Scores range from 0 to 1, with higher scores indicating more severe frailty.

**Figure 2.**
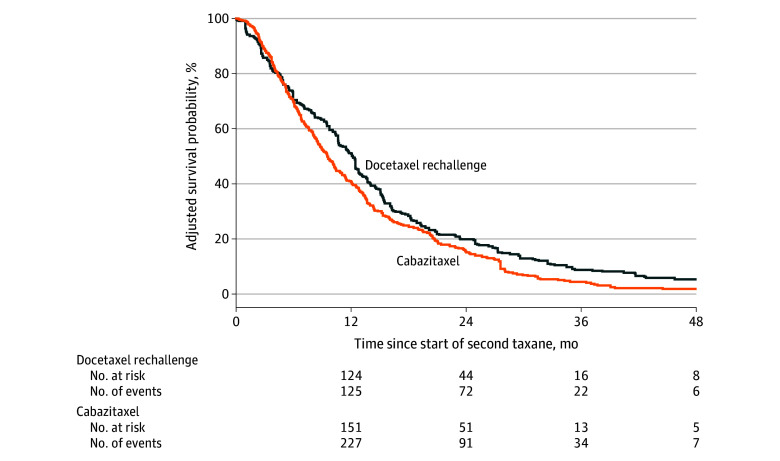
Weighted Kaplan-Meier Plot of Overall Survival in Patients Treated With Docetaxel Rechallenge and Cabazitaxel

**Figure 3.**
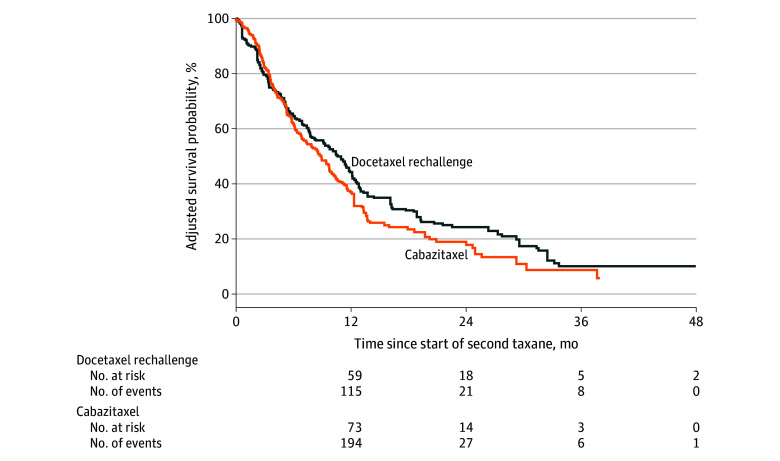
Weighted Kaplan-Meier Plot of the Time to Next Systemic Treatment or Death in Patients Treated With Docetaxel Rechallenge and Cabazitaxel

Patients treated with docetaxel rechallenge had a significantly longer OS from the start of the second course of taxane (median, 12.3 [IQR, 10.5-13.8] months) compared with cabazitaxel treatment (median, 9.6 [IQR, 8.6-11.1] months), with a hazard ratio of 0.81 (95% CI, 0.55-0.99; *P* = .04) (Figure 2). Descriptive analysis of secondary outcomes was consistent with this finding. Among patients treated with docetaxel rechallenge, a weighted 23.9 patients (9.8%) achieved a reduction of 90% or more from the baseline measurement taken before the second round of taxane therapy, while only a weighted 11.2 patients (3.0%) treated with cabazitaxel achieved a reduction of 90% or more (eTable 2 in Supplement 1). Time to subsequent systemic treatment or death was 10.7 (IQR, 7.8-12.7) months for the docetaxel rechallenge group and 8.9 (IQR, 7.1-10.5) months for the cabazitaxel group (Figure 3). The use of platinum, immunotherapy, and poly (ADP-ribose) polymerase inhibitors was similar between those treated with docetaxel rechallenge and cabazitaxel both in the period between taxane treatments (eTable 3 in Supplement 1) and after the second taxane treatment (eTable 4 in Supplement 1). The use of hormone therapy between mCRPC diagnosis and initial docetaxel therapy was also similar (eTable 5 in Supplement 1). A weighted 99.9 patients (37.7%) treated with docetaxel rechallenge and a weighted 158.6 patients (39.9%) treated with cabazitaxel received systemic therapies after the second taxane treatment.

The use of G-CSF drugs (filgrastim or pegfilgrastim) after the start of docetaxel rechallenge or cabazitaxel treatment was investigated. Overall, a weighted 173.3 patients (26.2%) received G-CSFs at least once, more often with cabazitaxel (46.1 patients [32.0%]) than docetaxel rechallenge (127.2 patients [17.4%]). A weighted 232.1 patients (35.0%) in both groups received therapies commonly used for neuropathy (ie, gabapentin, pregabalin, and/or duloxetine) at least once after the index date.

## Discussion

In this nationwide analysis of VA patients with mCRPC treated with docetaxel, docetaxel rechallenge was associated with improved OS compared with switching to cabazitaxel. Using IPTW, we adjusted for a broad set of confounders, leveraging comprehensive national data. Secondary outcome analyses were directionally consistent with the primary OS finding, supporting the robustness of this observation.

Taxanes became the standard of care in mCRPC following data showing improved survival for docetaxel compared with mitoxantrone (SWOG 99-16^16^ and TAX-327^17,18^) and later data showing cabazitaxel efficacy after docetaxel (TROPIC^19^). The FIRSTANA trial compared docetaxel and cabazitaxel as first-line chemotherapy and reported no difference in OS.^10^ However, the trial enrolled chemotherapy-naive patients, whereas our cohort included patients who had previously responded to docetaxel and had no disease progression at the time of retreatment.

This docetaxel-sensitive population more closely resembles treatment-responsive cohorts in other cancers, such as platinum-sensitive ovarian cancer in which retreatment with platinum elicits responses. Thus, our findings reflect a distinct clinical scenario and are not contradictory to those of the FIRSTANA study.

In clinical practice, breaks in treatment are often taken after durable responses or to manage toxic effects. Many patients who discontinue docetaxel in this way may later be considered for reinitiation, while others are switched to cabazitaxel despite no evidence of docetaxel resistance. This pragmatic pattern, especially within the VA system, provided the rationale for our study.

Our analysis focused on patients who received both a first and second line of taxane chemotherapy, excluding those previously treated with taxanes for metastatic hormone-sensitive disease to maintain cohort homogeneity. Notably, before IPTW, the docetaxel rechallenge group had less aggressive disease features at baseline (lower PSA levels and fewer visceral metastases) but worse general health (older age and higher levels of frailty). Cabazitaxel-treated patients had longer treatment intervals and received more prior therapies, including ARPIs. This suggests that docetaxel rechallenge may have been preferentially used for patients with greater frailty and more indolent disease, and cabazitaxel was used for fitter patients with more aggressive disease, thereby introducing potential selection bias. However, after IPTW, all these characteristics were similar in patients treated with docetaxel rechallenge and cabazitaxel , indicating that the weighting procedure successfully eliminated selection bias due to these factors.

Patients had received a median of 3 prior systemic therapies, and PSA response to initial docetaxel treatment (approximately 20%) was lower than expected. Their late use of a second taxane and high comorbidity burden likely contributed to shorter OS compared with trial populations.^8,9,20^

Although our primary hypothesis favored cabazitaxel, docetaxel rechallenge was associated with improved OS. Descriptive secondary end points (PSA response, time to next treatment) were also more favorable in the docetaxel rechallenge group. Use of subsequent therapies—including biomarker-based agents—was similar in both groups (39.9% vs 37.7%), supporting contemporaneous treatment approaches.

### Limitations

This study has some limitations. Although we used IPTW to successfully balance numerous measured confounders in an approach designed to emulate a randomized clinical trial, residual confounding is nevertheless possible for certain unmeasured confounders. For instance, while data on clinician-assessed performance status (eg, Eastern Cooperative Oncology Group score) were not available, our model included the validated VA Frailty Index, which estimates outcomes highly correlated with performance status.^21,22^ Symptom burden, tumor genomics (eg, *TP53*, *RB1*, *PTEN*), and pharmacogenomic factors (eg, *SLCO1B3*) were not available.^23^ Chemotherapy dosing data were also lacking, though FIRSTANA showed no OS difference between cabazitaxel doses of 25 and 20 mg/m^2^.^10^ Neuropathy may be underreported, as were dose modifications and supportive medications. Additionally, this study was conducted within the US VA health care system, and these findings may not fully generalize to broader populations. The VA cohort is older and has more comorbidities than typical trial populations, although its nationwide scope provides valuable clinical insight. VA data also do not capture treatments received outside the system if not paid for by the VA.

In this study, progression was assessed using PSA criteria only; however, in mCRPC, progression may also be defined by PSA, radiographic, or clinical measures per Prostate Cancer Working Group 3 criteria, which were not incorporated here. Only patients receiving 2 taxane lines were included, excluding many patients with mCRPC who do not receive second-line therapy. This limits generalizability to patients receiving 2 taxane lines. Moreover, the higher median number of intervening therapies in the cabazitaxel group may reflect more aggressive disease or a delayed switch strategy, but we accounted for this difference in the weighted analysis.

From a cost perspective, cabazitaxel is substantially more expensive than docetaxel and was associated with greater G-CSF use.^24,25^ There may also be further differences in costs of administration, monitoring, and supportive care between those drugs.^26^ While we did not perform a formal cost-effectiveness analysis, these observations suggest docetaxel rechallenge may be the more cost-conscious choice in appropriate candidates. In addition, patient preference might also influence the decision as to the use of taxane-based chemotherapy.^27^

Randomized clinical trials comparing docetaxel rechallenge and cabazitaxel in this setting are unlikely to be feasible. The treatment landscape is increasingly complex, with diverse prior therapies and evolving standards. Clinical equipoise is limited, particularly given the negative findings of the FIRSTANA trial. In this context, high-quality clinical data are essential to inform sequencing decisions.

Although the absolute survival difference of approximately 2.7 months appears modest, this magnitude is comparable to other meaningful gains seen in mCRPC. Docetaxel rechallenge may offer a tolerable, lower-cost option for patients previously responsive to taxane therapy, and the choice between agents should consider prior tolerance, toxicity profile, and patient preference. Current guidelines already list docetaxel rechallenge as a category 2A recommendation, and our results reinforce that position.

## Conclusions

In this cohort study of 669 patients with mCRPC, docetaxel rechallenge was associated with improved OS compared to cabazitaxel in patients who did not experience disease progression during prior docetaxel treatment. These findings support docetaxel rechallenge as a reasonable treatment option in appropriately selected patients when retreatment is clinically indicated.

## Notes

Supplement 1. **eTable 1.** Patient Characteristics in the Unweighted Cohort **eTable 2.** PSA Response to the Second Round of Taxanes in the Weighted Cohort of Patients Treated With Docetaxel Rechallenge (rDOC) and Cabazitaxel (CAB) **eTable 3.** Systemic Therapies Not Including Castration Received Between Taxanes in the Weighted Cohort of Patients Treated With Docetaxel Rechallenge (rDOC) and Cabazitaxel (CAB) **eTable 4.** Systemic Therapies Not Including Castration Received After the Index Date (Start of Second Round of Taxanes) in the Weighted Cohort of Patients Treated With Docetaxel Rechallenge (rDOC) and Cabazitaxel (CAB) **eTable 5.** Receipt of Hormone Therapy Between Metastatic Castrate-Resistant Prostate Cancer (mCRPC) Diagnosis and Start of Initial Docetaxel (DOC) in the Weighted Cohort of Patients Treated With Docetaxel Rechallenge (rDOC) and Cabazitaxel (CAB) **eFigure 1.** Standardized Mean Differences (SMD) of Covariates Before and After Inverse Probability of Treatment Weighting **eFigure 2.** Distribution of Propensity of Cabazitaxel Treatment Scores for Patients Treated With Cabazitaxel (CAB) vs Docetaxel Rechallenge (rDOC) in the Unweighted Cohort **eFigure 3.** Distribution of Inverse Probability of Treatment Weights for Patients Treated With Cabazitaxel (CAB) vs Docetaxel Rechallenge (rDOC) in the Unweighted Cohort

Supplement 2.
Data Sharing Statement
