# Supplementary material for: Docetaxel Rechallenge vs Cabazitaxel in Patients With Metastatic Castration-Resistant Prostate Cancer
Source: JAMA Netw Open. 2026 Jan 16;9(1):e2551231. doi: 10.1001/jamanetworkopen.2025.51231 (PMC12811811; doi:10.1001/jamanetworkopen.2025.51231)
Supplement: Supplement 1. — eTable 1. Patient Characteristics in the Unweighted Cohort eTable 2. PSA Response to the Second Round of Taxanes in the Weighted Cohort of Patients Treated With Docetaxel Rechallenge (rDOC) and Cabazitaxel (CAB) eTable 3. Systemic Therapies Not Including Castration Received Between Taxanes in the Weighted Cohort of Patients Treated With Docetaxel Rechallenge (rDOC) and Cabazitaxel (CAB) eTable 4. Systemic Therapies Not Including Castration Received After the Index Date (Start of Second Round of Taxanes) in the Weighted Cohort of Patients Treated With Docetaxel Rechallenge (rDOC) and Cabazitaxel (CAB) eTable 5. Receipt of Hormone Therapy Between Metastatic Castrate-Resistant Prostate Cancer (mCRPC) Diagnosis and Start of Initial Docetaxel (DOC) in the Weighted Cohort of Patients Treated With Docetaxel Rechallenge (rDOC) and Cabazitaxel (CAB) eFigure 1. Standardized Mean Differences (SMD) of Covariates Before and After Inverse Probability of Treatment Weighting eFigure 2. Distribution of Propensity of Cabazitaxel Treatment Scores for Patients Treated With Cabazitaxel (CAB) vs Docetaxel Rechallenge (rDOC) in the Unweighted Cohort eFigure 3. Distribution of Inverse Probability of Treatment Weights for Patients Treated With Cabazitaxel (CAB) vs Docetaxel Rechallenge (rDOC) in the Unweighted Cohort [file jamanetwopen-e2551231-s001.pdf]

Supplementary Online Content

Barata PC, Corrigan JK, La J, et al. Docetaxel rechallenge vs cabazitaxel in patients with metastatic castration-resistant prostate cancer. *JAMA Netw Open.* 2025;9(1):e2551231. doi:10.1001/jamanetworkopen.2025.51231

eTable 1. Patient Characteristics in the Unweighted Cohort
eTable 2. PSA Response to the Second Round of Taxanes in the Weighted Cohort of Patients Treated With Docetaxel Rechallenge (rDOC) and Cabazitaxel (CAB)
eTable 3. Systemic Therapies Not Including Castration Received Between Taxanes in the Weighted Cohort of Patients Treated With Docetaxel Rechallenge (rDOC) and Cabazitaxel (CAB)
eTable 4. Systemic Therapies Not Including Castration Received After the Index Date (Start of Second Round of Taxanes) in the Weighted Cohort of Patients Treated With Docetaxel Rechallenge (rDOC) and Cabazitaxel (CAB)
eTable 5. Receipt of Hormone Therapy Between Metastatic Castrate-Resistant Prostate Cancer (mCRPC) Diagnosis and Start of Initial Docetaxel (DOC) in the Weighted Cohort of Patients Treated With Docetaxel Rechallenge (rDOC) and Cabazitaxel (CAB)
eFigure 1. Standardized Mean Differences (SMD) of Covariates Before and After Inverse Probability of Treatment Weighting
eFigure 2. Distribution of Propensity of Cabazitaxel Treatment Scores for Patients Treated With Cabazitaxel (CAB) vs Docetaxel Rechallenge (rDOC) in the Unweighted Cohort
eFigure 3. Distribution of Inverse Probability of Treatment Weights for Patients Treated With Cabazitaxel (CAB) vs Docetaxel Rechallenge (rDOC) in the Unweighted Cohort

This supplementary material has been provided by the authors to give readers additional information about their work.

© 2026 Barata PC et al. *JAMA Network Open*.

eTable 1. Patient Characteristics in the Unweighted Cohort

	Overall	rDOC	CAB	SMD
n	669	262	407	
Age, median [IQR]	72.18 [67.49, 76.84]	73.63 [68.38, 79.18]	71.53 [66.99, 75.76]	0.353
Race and ethnicity, n (%)				0.063
Black	196 (29.3)	79 (30.2)	117 (28.7)	
Hispanic	29 (4.3)	10 (3.8)	19 (4.7)	
Non-Hispanic White	407 (60.8)	160 (61.1)	247 (60.7)	
Other/Unknown	37 (5.5)	13 (5.0)	24 (5.9)	
Time of metastatic disease diagnosis, n (%)				0.196
Synchronous	225 (33.6)	74 (28.2)	151 (37.1)	
Metachronous	246 (36.8)	101 (38.5)	145 (35.6)	
Unknown	198 (29.6)	87 (33.2)	111 (27.3)	
Stage at initial docetaxel, n (%)				0.243
Pelvic lymph nodes ONLY (N1)	27 (4.0)	13 (5.0)	14 (3.4)	
Lymph nodes outside pelvis (M1a)	52 (7.8)	21 (8.0)	31 (7.6)	
Bone with or w/o lymph node (M1b)	506 (75.6)	199 (76.0)	307 (75.4)	
Visceral (liver, lung, brain) with or w/o bone or lymph node (M1c)	69 (10.3)	19 (7.3)	50 (12.3)	
Unknown	15 (2.2)	10 (3.8)	5 (1.2)	
Stage at index date, n (%)				0.346
Pelvic lymph nodes ONLY (N1)	17 (2.5)	10 (3.8)	7 (1.7)	
Lymph nodes outside pelvis (M1a)	32 (4.8)	16 (6.1)	16 (3.9)	
Bone with or w/o lymph node (M1b)	499 (74.6)	202 (77.1)	297 (73.0)	
Visceral (liver, lung, brain) with or w/o bone or lymph node (M1c)	115 (17.2)	29 (11.1)	86 (21.1)	
Unknown	6 (0.9)	5 (1.9)	1 (0.2)	
PSA doubling time at index date, n (%)				0.206

© 2026 Barata PC et al. *JAMA Network Open*.

Greater than 3 mo	175 (26.2)	75 (28.6)	100 (24.6)	
Less than 3 mo	470 (70.3)	183 (69.8)	287 (70.5)	
Unknown	24 (3.6)	4 (1.5)	20 (4.9)	
PSA at index date, median [IQR]	76.91 [21.15, 259.00]	44.25 [11.06, 163.22]	96.15 [35.17, 309.65]	0.219
Gleason score of median or above, n (%)	598 (89.4)	210 (80.2)	388 (95.3)	0.476
Cycles of initial DOC, median [IQR]	6.00 [4.00, 10.00]	6.00 [4.00, 9.00]	7.00 [5.00, 10.00]	0.192
PSA response to initial DOC, n (%)				0.199
>=50% decline	125 (18.7)	53 (20.2)	72 (17.7)	
>=70% decline	70 (10.5)	35 (13.4)	35 (8.6)	
>=90% decline	16 (2.4)	8 (3.1)	8 (2.0)	
Stable PSA	458 (68.5)	166 (63.4)	292 (71.7)	
Number of systemic therapies before initial DOC, median [IQR]	1.00 [0.00, 2.00]	1.00 [0.00, 2.00]	1.00 [0.00, 2.00]	0.008
Number of systemic therapies between mCRPC diagnosis and index, median [IQR]	2.00 [1.00, 2.00]	1.50 [1.00, 2.00]	2.00 [1.00, 2.00]	0.337
Number of systemic therapies between taxanes, median [IQR]	1.00 [0.00, 1.00]	0.00 [0.00, 1.00]	1.00 [0.00, 1.00]	0.407
Time from PCa diagnosis to index in months, median [IQR]	90.28 [49.64, 150.02]	97.35 [50.37, 162.55]	85.48 [49.50, 144.97]	0.149
Time from initial DOC to index in months, median [IQR]	11.24 [7.86, 16.80]	9.50 [6.88, 15.08]	12.39 [9.17, 18.25]	0.234
Comorbidities, n (%)				
Alzheimers disease	2 (0.3)	0 (0.0)	2 (0.5)	0.099
Anemia	363 (54.3)	146 (55.7)	217 (53.3)	0.048
Chronic kidney disease	273 (40.8)	111 (42.4)	162 (39.8)	0.052
Cardiovascular disease	175 (26.2)	75 (28.6)	100 (24.6)	0.092
Diabetes	244 (36.5)	105 (40.1)	139 (34.2)	0.123
Liver disease or viral hepatitis	100 (14.9)	39 (14.9)	61 (15.0)	0.003
VA Frailty Index, median [IQR]	0.23 [0.16, 0.32]	0.26 [0.19, 0.35]	0.23 [0.16, 0.32]	0.254
Albumin, n (%)				0.117

© 2026 Barata PC et al. *JAMA Network Open*.

Greater than 3.5 g/dL	422 (63.1)	160 (61.1)	262 (64.4)	
Less than 3.5 g/dL	198 (29.6)	78 (29.8)	120 (29.5)	
Unknown	49 (7.3)	24 (9.2)	25 (6.1)	
Hemoglobin, n (%)				0.189
Greater than 10 g/dL	491 (73.4)	202 (77.1)	289 (71.0)	
Less than 10 g/dL	158 (23.6)	50 (19.1)	108 (26.5)	
Unknown	20 (3.0)	10 (3.8)	10 (2.5)	
LDH, n (%)				0.148
High LDH	127 (19.0)	44 (16.8)	83 (20.4)	
Not High LDH	59 (8.8)	29 (11.1)	30 (7.4)	
Unknown	483 (72.2)	189 (72.1)	294 (72.2)	
Alkaline Phosphatase, n (%)				0.441
Greater than 130	244 (36.5)	63 (24.0)	181 (44.5)	
Less than 130	403 (60.2)	189 (72.1)	214 (52.6)	
Unknown	22 (3.3)	10 (3.8)	12 (2.9)	

Indicates before inverse probability of treatment weighting in the cohort of patients treated with docetaxel rechallenge (rDOC) or cabazitaxel (CAB).

© 2026 Barata PC et al. *JAMA Network Open*.

eTable 2. PSA Response to the Second Round of Taxanes in the Weighted Cohort of Patients Treated With Docetaxel Rechallenge (rDOC) and Cabazitaxel (CAB)

	Overall	rDOC	CAB
n	662.4	265.2	397.2
PSA Maximum Decline, n (%)			
>= 30% decline	222.4 (36.3)	102.8 (42.0)	119.6 (32.5)
>= 50% decline	143.7 (23.4)	65.9 (26.9)	77.8 (21.1)
>= 90% decline	35.1 (5.7)	23.9 (9.8)	11.2 (3.0)
Stable	196.0 (32.0)	73.7 (30.1)	122.3 (33.2)
No Decline	195.0 (31.8)	68.4 (27.9)	126.6 (34.4)

© 2026 Barata PC et al. *JAMA Network Open*.

eTable 3. Systemic Therapies Not Including Castration Received Between Taxanes in the Weighted Cohort of Patients Treated With Docetaxel Rechallenge (rDOC) and Cabazitaxel (CAB)

	Overall	rDOC	CAB
n	348.1	117.8 (38.0)	230.2 (62.0)
Systemic Therapies, n (%)			
ARPIs	312.9 (58.3)	106.0 (49.5)	206.9 (64.1)
Carboplatin	15.8 (2.9)	10.0 (4.7)	5.8 (1.8)
PARP inhibitors	12.1 (2.2)	1.8 (0.9)	10.2 (3.2)
Pembrolizumab	7.3 (1.4)	0.0 (0.0)	7.3 (2.2)

Patients who did not receive any systemic therapies between taxanes are not shown.

© 2026 Barata PC et al. *JAMA Network Open*.

eTable 4. Systemic Therapies Not Including Castration Received After the Index Date (Start of Second Round of Taxanes) in the Weighted Cohort of Patients Treated With Docetaxel Rechallenge (rDOC) and Cabazitaxel (CAB)

	Overall	rDOC	CAB
n	258.5	99.9 (35.0)	158.6 (61.4)
Systemic Therapies, n (%)			
ARPIs	167.0 (64.6)	72.2 (72.3)	94.8 (59.8)
Carboplatin	60.5 (23.4)	17.2 (17.2)	43.3 (27.3)
Cabazitaxel	4.8 (1.9)	4.8 (4.8)	0.0 (0.0)
PARP inhibitors	39.4 (15.2)	14.8 (14.8)	24.6 (15.5)
Pembrolizumab	12.8 (4.9)	3.6 (3.6)	9.1 (5.8)

Patients who did not receive any systemic therapies after the index date are not shown.

© 2026 Barata PC et al. *JAMA Network Open*.

eTable 5. Receipt of Hormone Therapy Between Metastatic Castrate-Resistant Prostate Cancer (mCRPC) Diagnosis and Start of Initial Docetaxel (DOC) in the Weighted Cohort of Patients Treated With Docetaxel Rechallenge (rDOC) and Cabazitaxel (CAB)

	Overall	rDOC	CAB
Hormone Therapy, n (%)	516.9 (94.2)	212.6 (94.5)	304.3 (93.9)

© 2026 Barata PC et al. *JAMA Network Open*.

eFigure 1. Standardized Mean Differences (SMD) of Covariates Before and After Inverse Probability of Treatment Weighting

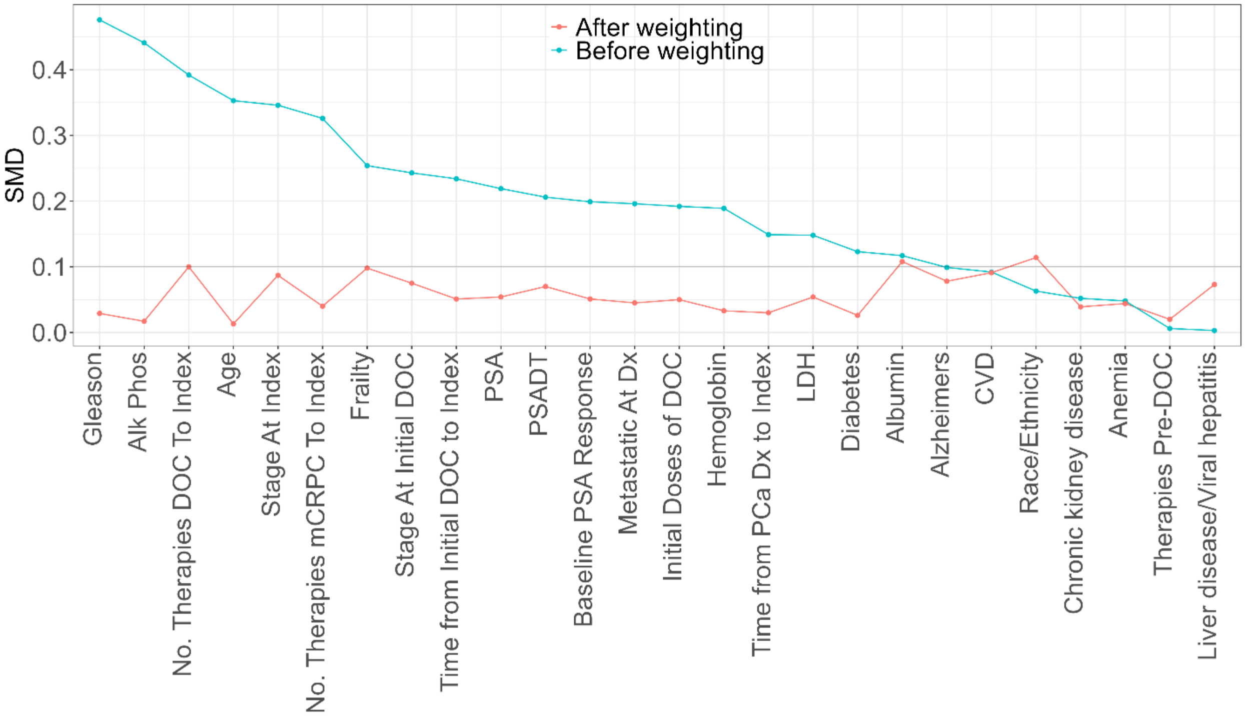

© 2026 Barata PC et al. *JAMA Network Open*.

eFigure 2. Distribution of Propensity of Cabazitaxel Treatment Scores for Patients Treated With Cabazitaxel (CAB) vs Docetaxel Rechallenge (rDOC) in the Unweighted Cohort

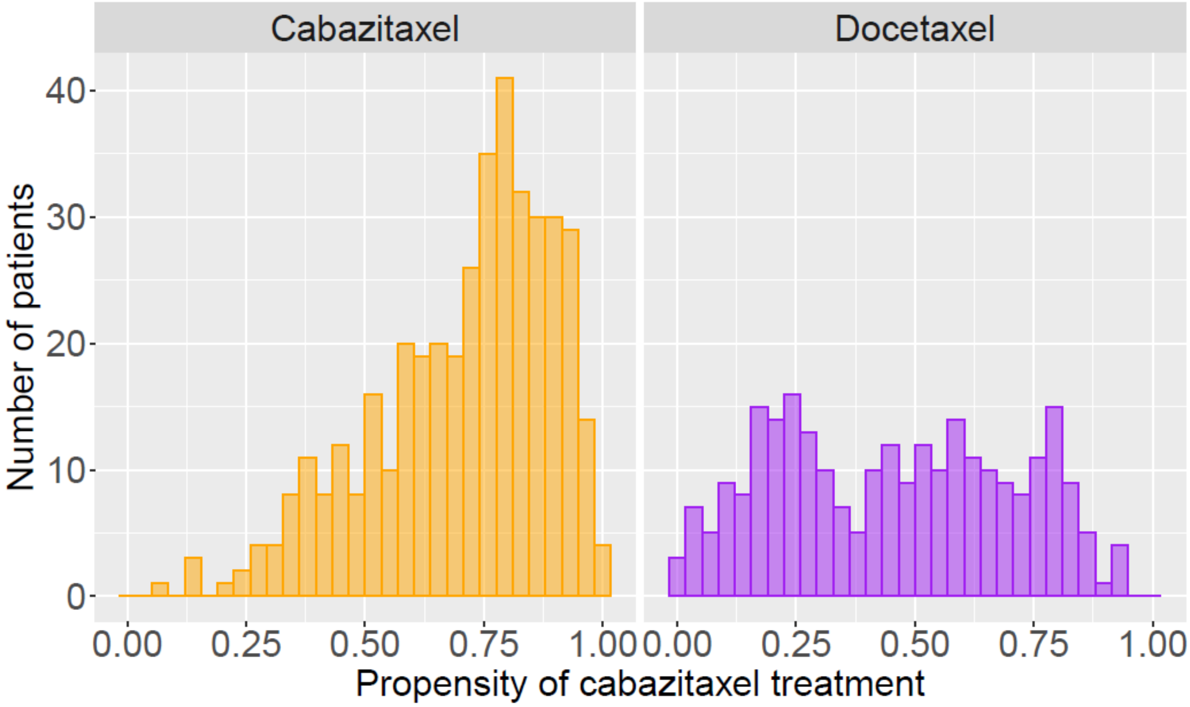

© 2026 Barata PC et al. *JAMA Network Open*.

eFigure 3. Distribution of Inverse Probability of Treatment Weights for Patients Treated With Cabazitaxel (CAB) vs Docetaxel Rechallenge (rDOC) in the Unweighted Cohort

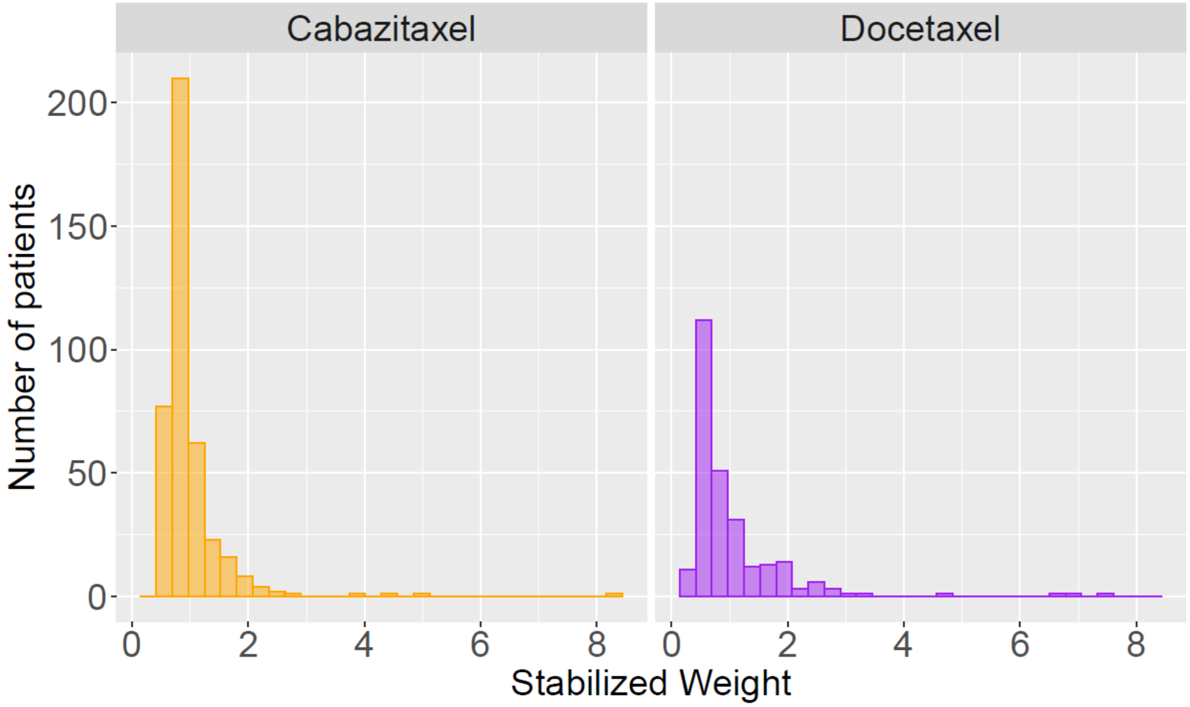

© 2026 Barata PC et al. *JAMA Network Open*.
