# Supplementary material for: Docetaxel Rechallenge vs Cabazitaxel in Patients With Metastatic Castration-Resistant Prostate Cancer
Source: JAMA Netw Open. 2026 Jan 16;9(1):e2551231. doi: 10.1001/jamanetworkopen.2025.51231 (PMC12811811; doi:10.1001/jamanetworkopen.2025.51231)
Supplement: Supplement 2. — Data Sharing Statement [file jamanetwopen-e2551231-s002.pdf]

Data Sharing Statement

Barata. Docetaxel Rechallenge vs Cabazitaxel in Patients With Metastatic Castration-Resistant Prostate Cancer. *JAMA Netw Open*. Published January 16, 2026.
doi:10.1001/jamanetworkopen.2025.51231

Data
Data available: No

Additional Information
Explanation for why data not available: The data that support the findings of this study are available from the VA. VA data are made freely available to researchers behind the VA firewall with an approved VA study protocol. More information is available at https://www.virec.research.va.gov or the VA Information Resource Center (VIReC) at VIReC@va.gov.
